# Influence of Tip Diameter and Light Spectrum of Curing Units on the Properties of Bulk-Fill Resin Composites

**DOI:** 10.1055/s-0041-1735799

**Published:** 2021-12-14

**Authors:** Igor Oliveiros Cardoso, Alexandre Coelho Machado, Luísa de Oliveira Fernandes, Paulo Vinícius Soares, Luís Henrique Araújo Raposo

**Affiliations:** 1 Department of Occlusion, Fixed Prosthodontics and Dental Materials, School of Dentistry, Federal University of Uberlândia, Uberlândia, Brazil; 2 Technical School of Health, Federal University of Uberlândia, Uberlândia, Brazil; 3 Department of Operative Dentistry and Dental Materials, School of Dentistry, Federal University of Uberlândia, Uberlândia, Brazil

**Keywords:** resin composite, light curing, polymerization

## Abstract

**Objective** The aim of this study was to evaluate the influence of different light-curing units (LCUs) with distinct tip diameters and light spectra for activating bulk-fill resins.

**Materials and Methods** The specimens ( *n* = 10) were made from a conventional composite (Amaris, VOCO) and bulk-fill resins (Aura Bulk Fill, SDI; Filtek One, 3M ESPE; Tetric Bulk Fill, Ivoclar Vivadent) with two diameters, 7 or 10 mm, × 2 mm thickness. Following 24 hours of specimen preparation, the degree of conversion (DC) was evaluated using the Fourier-transform infrared unit. Knoop hardness (KHN) readings were performed on the center and periphery of the specimens. Data were assessed for homoscedasticity and submitted to one-way and three-way analysis of variance followed by the Tukey's and Dunnett's tests, depending on the analysis performed (α = 0.05).

**Results** LCUs and specimen diameter significantly affected the DC. The *Tetric Bulk Fill* provided increased DC results when light-cured with *Valo* (54.8 and 53.5%, for 7 and 10 mm, respectively) compared with *Radii Xpert* (52.1 and 52.9%, for 7 and 10 mm, respectively). No significant differences in KHN results were noted for the conventional resin composite ( *Amaris* ) compared with LCUs ( *p* = 0.213) or disc diameters ( *p* = 0.587), but the center of the specimen exhibited superior KHN ( *p* ≤ 0.001) than the periphery.

**Conclusion** The light spectrum of the multipeak LCU ( *Valo* ) significantly increased the DC and KHN of the bulk-fill resin composite with additional initiator to camphorquinone ( *Tetric Bulk Fill* ) compared with the monowave LCU ( *Radii Xpert* ). The tip size of the LCUs influenced the performance of some of the resin composites tested.

## Introduction

Resin-based composites (RBCs) are widely used materials for Classes I and II restorations with failure rates of 1.8% after 5 years and 2.4% after 10 years. 1 Although conventional RBCs exhibit good mechanical properties, they also present undesirable characteristics, such as polymerization shrinkage. 2 This shrinkage results in residual stress in the tooth-restoration interface. If not controlled or reduced either by the operator or material, the shrinkage stress is related to marginal staining, enamel cracks, and postoperative sensitivity. 2 According to the World Dental Federation, direct restorations can fail based on aesthetic, functional, or biological aspects. 3 Shrinkage stress may be related with all these criteria regardless of whether manifested early or in late stages, leading to failures. 3 Some measures can be taken to reduce the influence of polymerization shrinkage; for example, the use of incremental insertion techniques or bulk-fill composite materials would be beneficial for the final restoration. 4 Beyond decreasing the clinical time of the restorative procedure, bulk-fill resin composites are used in single increments of up to 4 or 5 mm thickness because they present lower polymerization shrinkage and consequently lower residual shrinkage stress. 5

For RBCs restorations to be successful and acquire adequate mechanical and optical properties, proper polymerization is required. 6 Some relevant properties for the success of a restoration, such as the degree of conversion (DC) and hardness, are influenced by irradiance and the light spectrum. 6 7 The DC, namely, monomers converting to polymers, is directly related to hardness, a property that expresses the mechanical and wear resistance of resin-based composites. 5 7 The light-curing unit (LCU) provides the light that will allow the activation of initiators present in the composites to trigger the polymerization process. 7 Currently, the most widely used LCUs include light-emitting diodes (LED) that can present different spectra, and these devices are classified into monowave and multipeak units. 8 Monowave LED units present a light spectrum between 450 and 490 nm. This light spectrum is effective in activating the camphorquinone (CQ) initiator, which has its peak action at 468 nm and is the most commonly used agent in resin-based materials. 9 Multipeak LED units present violet light in addition to blue light with emission of wavelengths below 420 nm, allowing the activation of different initiators. 9

The light tips of the LCUs have different diameters, 10 and their sizes often do not coincide with the mesiodistal distances (MD-Ds) of the posterior teeth, which ranges from 6.74 to 7.16 mm in premolars and from 9.72 to 11.03 mm in molars. 11 For the incremental technique, this factor may not be relevant given that each increment must be individually activated by light. However, for the use of bulk-fill resins, only one light-activation cycle is typically performed. Under these circumstances, the MD-D from the teeth and the diameter of the LED tip must be known to perform proper light curing to the whole restoration and consequently allow for sufficient polymerization. 12 LCUs with small tip diameter used to activate large molar restorations may not completely cover the resin composite, potentially resulting in insufficient polymerization. 7

Thus, the aim of this study was to evaluate the influence of different LED-based LCUs with different tip diameters and light spectra for activating bulk-fill RBCs. The null hypothesis generated was that LCUs with different tip sizes and light spectra would not influence the DC and Knoop hardness (KHN) of different bulk-fill RBCs.

## Materials and Methods

### Irradiance Measurement

The curing units, Valo (Ultradent, Salt Lake City, Utah, United States) and Radii Xpert (SDI, Bayswater, Australia) were fully charged as recommended by the manufacturer. The higher power (mW) of the cordless LED units during the cycle was individually checked for five light cycles of 20 seconds using a power meter (Nova, Ophir Spiricon, Logan, Utah, United States), then the average of the five cycles was divided by the tip area (cm ^2^ ), calculated from the optical diameter, as measured with a digital caliper (CD6CS, Mitutoyo, Kanagawa, Japan), to obtain the irradiance (mW/cm ^2^ ) ( Table 1 ). 13

**Table 1:** Specifications of the LCU tested

LCU	Manufacturer	Irradiance	Wavelength emission	Tip diameter (mm) 18	Tip area (cm ^2^ ) 18
Radii Xpert	SDI, Bayswater, Victoria, Australia	1,575 mW/cm ^2^ (standard)	Monowave	7.8	0.48
Valo	Ultradent, Salt Lake City, Utah, United States	1103 mW/cm ^2^ (standard)	Multipeak	9.5	0.7

Abbreviation: LCU, light-curing unit.

### Specimen Preparation

To simulate the diameter of average occlusal cavities in premolars and molars, 2-mm-thick resin cylinders with 7 and 10 mm diameter, respectively, were made from the conventional and bulk-fill composites as described in Table 2 . For this, circular aluminum matrixes were positioned over glass plates, and specimens were obtained by inserting the RBCs in a single increment. Then, a Mylar strip and a glass plate were placed over the resin and slightly compressed to regularize the top surface of the specimens.

**Table 2:** Specifications of the tested RBCs

Resin composite	Manufacturer	Color	Type	Organic matrix	Filler	Amount of load (wt%/vol%)	Batch no.
Amaris	VOCO, Cuxhaven, Germany	TN	Conventional Nanohybrid	Bis-GMA, UDMA, TEGDMA	Inorganics fillers in a methacrylate matrix	80/–	1829623
Filtek One	3M ESPE, St. Paul, Minnesota, United States	A2	Bulk fill	AFM, AUDMA, UDMA, and 1,2-dodecano-DMA (DDMA)	Ytterbium trifluoride, nonaggregated silica, nonaggregated zirconia, zirconia/silica clusters	76.5/58	N974887
Aura Bulk Fill	SDI, Bayswater, Victoria, Australia	BKF	Bulk fill	Bis-GMA, UDMA, Bis-EMA, TEGDMA	Silica, signaled barium and glass particles	74.2/65	180143
Tetric Bulk Fill	Ivoclar Vivadent, Schaan, Liechtenstein	IVA	Bulk fill	Bis-GMA, BisEMA and UDMA	Barium aluminum silicate glass, an “Isofiller,” ytterbium fluoride and spherical mixed oxide	75–77/55	94624

Abbreviation: RBCs, resin-based composites.

For light curing the specimens, the tip of the LCU ( Table 1 ) was positioned parallel and in close contact to the top glass plate, and light curing was performed for 20 seconds, as recommended by each composite manufacturer, using a standardized position. The position of each specimen in relation to the LCU tip was noted in the top of the discs with permanent marker to allow the same position to be determined during the tests. After this, the specimens were stored under dry conditions in identified light-proof containers.

### Degree of Conversion

Twenty-four hours after specimen preparation, the DC was evaluated at the center of the top surface of the specimens ( *n* = 10) using attenuated total reflectance Fourier-transform infrared (FTIR) spectroscopy unit (Tensor 27, Bruker, Ettlingen, Germany). To determine the number of carbon bonds remaining, a percentage was obtained between the aliphatic C = C (vinyl) (1,638 cm ^−1^ ) and aromatic C = C absorption (1,608 cm ^−1^ ) chains for both cured and uncured specimens. The spectra of cured and uncured specimens were obtained using 32 scans at 4 cm ^−1^ resolution within 1,000 to 6,000 cm ^−1^ range. The spectra were subtracted from the background spectra using the FTIR unit provided software (OMNIC 6.1, Nicolet 138 Instrument Corp, Madison, Wisconsin, United States). The DC was calculated using the following equation: DC (%) = (1− [cured aliphatic/aromatic ratio]/[uncured aliphatic/144 aromatic ratio]) × 100. 6

### Knoop Hardness

KHN specimens were included in polyester resin to allow for better handling during polishing and hardness tests. Then, the specimens were submitted to sequential wet polishing using sandpapers (#100, 600, 1,200, 2,000, and 3,000 grit; 3M, Sumaré, São Paulo, Brazil) in an automatized polisher for 1 minute in each polisher. Sequentially, the specimens received final polishing using felt discs associated with 1 and 0.25 µm metallographic diamond pastes (Arotec, Cotia, São Paulo, Brazil) for 1 minute in each polisher. The specimens were then washed with deionized water.

After air-drying, the specimens were submitted to KHN tests (HMV-2; Shimadzu, Kyoto, Japan), which were performed on the top surface by applying a load of 100 g for 10 seconds. Fifteen indentations were performed in each specimen at five different areas with 3 indentations in the central area and 12 in the periphery with 3 in each extremity: superior, inferior, left, and right, 1 mm away from the margin of the disc. The KHN corresponding to each indentation was determined by measuring the dimensions of the indentation using the following formula: KHN = 14.2 ( *F* = *d/d* ^2^ ), where *F* is the test load in kg, and *d* is the longer diagonal length of an indentation in mm. Then, the KHN value was determined by obtaining the arithmetic mean of indentations made in the center and peripheries. 5

## Statistical Analysis

The data collected for DC and KHN were assessed for homoscedasticity and submitted to three-way analysis of variance (ANOVA). Multiple comparisons were made using the Tukey's test within the experimental groups. One-way ANOVA followed by Dunnett's test was used for comparisons between control and experimental groups. All the tests were conducted at an α = 0.05 significance level. The analyses were performed using a statistical software (SigmaPlot 12.0, Systat Software, San Jose, California, United States).

## Results

### Degree of Conversion

The DC results are shown in Tables 3 and 4 . *Tetric Bulk Fill* exhibited increased DC compared with conventional resin composite for both diameters and LCUs evaluated. For *Filtek One* , significant differences from the control group were only observed for 10-mm specimens light-cured with *Radii Xpert* , which presented increased DC. *Aura Bulk Fill* exhibited increased DC compared with the control group in almost all conditions. However, no significant differences were verified for 10-mm specimens light-cured with *Valo* . None of the bulk-fill RBCs exhibited significantly reduced DC results compared with the control group. In most situations, bulk-fill RBCs exhibited superior or statistically similar DC results ( Table 3 ).

**Table 3:** Mean DC% values and standard deviation (±) for control and experimental groups according to LCU and specimen diameter

Group	Diameter	LCU	DC%	*p* -Value	LCU	DC%	*p* -Value
Amaris (CG)	7 mm	Valo	47.2 ± 3.6	–	Radii Xpert	45.9 ± 3.4	–
	10 mm		48.6 ± 3.6	–		44.9 ± 3.0	–
Aura Bulk Fill	7 mm		51.2 ± 1.9 a	0.004		51.7 ± 3.0 a	<0.001
	10 mm		50.4 ± 2.3	0.344		52.9 ± 2.9 a	<0.001
Filtek One	7 mm		49.7 ± 2.7	0.099		47.0 ± 2.0	0.753
	10 mm		49.6 ± 2.8	0.741		52.4 ± 2.2 a	<0.001
Tetric Bulk Fill	7 mm		54.8 ± 1.7 a	<0.001		52.1 ± 3.1 a	<0.001
	10 mm		53.5 ± 1.5 a	<0.001		52.9 ± 2.4 a	<0.001

Abbreviations: CG, control group; DC, degree of conversion; LCU, light-curing unit.

a Indicates significant difference from CG; one-way analysis of variance and Dunnett's test ( *p* > 0.05).

**Table 4:** Mean DC% and standard deviation (±) for bulk-fill RBCs according to LCU and specimen diameter

Group	Valo		Radii Xpert	
	7 mm	10 mm	7 mm	10 mm
Aura Bulk Fill	51.2 ± 1.9 Ab€	50.4 ± 2.3 Ab€	51.7 ± 3.0 Aa€	52.9 ± 2.9 Aa€
Filtek One	49.7 ± 2.7 Ab£	49.6 ± 2.8 Ab€	47.0 ± 2.0 Ab£	52.4 ± 2.2 Ab€
Tetric Bulk Fill	54.8 ± 1.7 Aa€	53.5 ± 1.5 Aa€	52.1 ± 3.1 Ba€	52.9 ± 2.4 Ba€

Abbreviations: DC, degree of conversion; LCU, light-curing unit; RBCs, resin-based composites.

Note: Capital letters indicate significant differences among LCUs (rows: vertical direction). Lowercase letters indicate significant differences among bulk-fill RBCs (columns: horizontal direction), and symbols indicate significant differences between diameters for the same LCU (rows: vertical direction). Tukey's test ( *p* ˂ 0.05).

LCUs and specimen diameter significantly affected DC results compared with bulk-fill RBCs ( Table 4 ). The *Tetric Bulk Fill* showed increased DC results (54.8 and 53.5% for 7 and 10 mm, respectively) when light-cured with *Valo* compared with *Radii Xpert* (52.1 and 52.9%, respectively). When using *Valo* , *Tetric Bulk Fill* also presented superior DC results compared with the other bulk-fill RBCs evaluated. The *Tetric Bulk Fill* and *Aura Bulk Fill* presented superior DC results compared with *Filtek One* when light curing with *Radii Xpert* . Significant differences were observed for DC results for the different specimen diameters in the *Filtek One* group.

### Knoop Hardness

The KHN results are described in Tables 5 and 6 . No significant differences were noted in KHN results for the conventional resin composite ( *Amaris* ) when comparing with LCUs ( *p* = 0.213) or disc diameters ( *p* = 0.587), but the center of the specimen exhibited superior KHN ( *p* ≤ 0.001) compared with periphery. KHN results for *Aura Bulk Fill* were not influenced by LCUs ( *p* = 0.049), specimen diameter ( *p* = 0.468), or region of analysis ( *p* = 0.083). For *Filtek On* e, similar KHN results were verified for the different LCUs ( *p* = 0.276), but 7-mm diameter specimens exhibited greater KHN than 10-mm diameter specimens ( *p* = 0.002), and the center region exhibited superior results compared with periphery ( *p* = 0.038). For *Tetric Bulk Fill* , light curing with *Valo* resulted in superior KHN compared with *Radii Xpert* ( *p* ≤ 0.001), and 7-mm specimens also presented increased KHN compared with 10-mm diameter specimens ( *p* = 0.015), but no significant differences were observed for the region of analysis.

**Table 5:** Mean KHN values and standard deviation (±) for control and experimental groups according to LCU, specimen diameter, and region of analysis

Group	Diameter	Region	LCU	KHN	*p* -Value	LCU	KHN	*p* -Value
Amaris (CG)	7 mm	Center	Valo	53.0 ± 4.4	–	Radii Xpert	51.3 ± 1.0	–
		Periphery		51.8 ± 3.7	–		48.8 ± 0.8	–
	10 mm	Center		53.2 ± 1.8	–		54.3 ± 1.7	–
		Periphery		57.6 ± 1.6	–		49.4 ± 1.4	–
Aura Bulk Fill	7 mm	Center		57.6 ± 2.5 a	0.032		58.8 ± 2.3 a	<0.001
		Periphery		55.3 ± 2.2	0.067		57.0 ± 1.9 a	<0.001
	10 mm	Center		58.3 ± 2.1 a	<0.001		57.5 ± 1.7 a	<0.001
		Periphery		57.6 ± 1.6 a	<0.001		57.4 ± 3.0 a	<0.001
Filtek One	7 mm	Center		70.5 ± 0.8 a	<0.001		69.9 ± 1.7 a	<0.001
		Periphery		69.4 ± 1.1 a	<0.001		69.7 ± 0.8 a	<0.001
	10 mm	Center		68.9 ± 2.1 a	<0.001		69.0 ± 1.1 a	<0.001
		Periphery		68.5 ± 2.0 a	<0.001		66.8 ± 1.0 a	<0.001
Tetric Bulk Fill	7 mm	Center		62.1 ± 0.9 a	<0.001		59.7 ± 1.7 a	<0.001
		Periphery		61.7 ± 0.7 a	<0.001		58.6 ± 1.2 a	<0.001
	10 mm	Center		60.6 ± 1.0 a	<0.001		58.1 ± 2.6 a	<0.001
		Periphery		60.0 ± 0.8 a	<0.001		57.4 ± 1.9 a	<0.001

Abbreviations: CG, control group; KHN, Knoop hardness; LCU, light-curing unit.

a Indicates significant difference from CG; one-way analysis of variance and Dunnett's test ( *p* > 0.05).

**Table 6:** Mean Knoop hardness and standard deviation (±) for bulk-fill RBCs according to LCU, specimen diameter and region of analysis

Group	Diameter	Valo		Radii Xpert	
		Center	Periphery	Center	Periphery
Amaris	7 mm	53.0 ± 4.4 Aa£	51.8 ± 3.7 Ba£	51.3 ± 1.0 Aa£	48.8 ± 0.8 Ba£
	10 mm	53.2 ± 1.8 Aa£	49.6 ± 1.5 Ba£	54.3 ± 1.7 Aa£	49.4 ± 1.4 Ba£
Aura Bulk	7 mm	57.6 ± 2.5 Aa£	55.3 ± 2.2 Aa£	58.8 ± 2.3 Aa£	57.0 ± 1.9 Aa£
	10 mm	58.3 ± 2.1 Aa£	57.6 ± 1.6 Aa£	57.5 ± 1.7 Aa£	57.4 ± 3.0 Aa£
Filtek One	7 mm	70.5 ± 0.8 Aa£	69.4 ± 1.1 Ba£	69.9 ± 1.7 Aa£	69.7 ± 0.8 Aa£
	10 mm	68.9 ± 2.1 Ab£	68.5 ± 2.0 Bb£	69.0 ± 1.1 Ab£	66.8 ± 1.0 Ab£
Tetric Bulk	7 mm	62.1 ± 0.9 Aa£	61.7 ± 0.7 Aa£	59.7 ± 1.7 Aa€	58.6 ± 1.2 Aa€
	10 mm	60.6 ± 1.0 Ab£	60.0 ± 0.8 Ab£	58.1 ± 2.6 Ab€	57.4 ± 1.9 Ab€

Abbreviations: DC, degree of conversion; LCU, light-curing unit; RBCs, resin-based composites.

Note: Capital letters indicate significant differences between center and periphery regions (rows: vertical direction). Lowercase letters indicate significant differences between disc diameters (columns: horizontal direction), and symbols indicate significant differences between LCUs for the same region (rows: vertical direction). Tukey's test ( *p* ˂ 0.05).

None of the experimental groups showed significantly reduced KHN results compared with the control group (Amaris). The 7-mm *Aura Bulk Fill* specimens photoactivated with *Valo* were not statistically different compared with *Amaris* (control group). All other groups presented significantly superior KHN results compared with the control group ( Table 5 ).

## Discussion

The LCUs tested in the present study present different tip diameters and light spectra and have influenced the DC and KHN of the bulk-fill RBCs tested. Thus, the null hypothesis tested was rejected.

The use of bulk-fill RBCs have increased substantially in recent years, and adequate light curing is essential to achieve the best mechanical properties with these materials. 7 The polymerization process of light-cured composites is completely dependent on the technical characteristics of the LCU, such as irradiance, wavelength range, diameter of the tip, and others. 14 Different LCUs can result in distinct physical properties for the same material given that the DC and hardness of RBCs may be affected as demonstrated by the results of this investigation and previous studies. 15 16

Different mechanisms can be used to allow deeper polymerization and reduced stress for bulk-fill composites. Some manufacturers achieve deeper polymerization by using additional or different photoinitiators, such as diphenyl phosphine oxide (Lucerin—TPO) or bis-(4-methoxybenzoyl)diethyl-germane (Ivocerin). 17 The properties of bulk-fill resins may also be improved when increased light transmission through the composite is possible, which its commonly achieved by changing the filler content. The presence of pigments and refractive index mismatch between the organic matrix and fillers are the main factors causing reduction in light transmission. 18

In the present study, no bulk-fill RBCs presented lower DC values than the conventional composite (control group). The LCU factor was only relevant for *Tetric N-Ceram Bulk Fill* , and this may be explained by the fact that this material has an additional initiator to CQ, Ivocerin, which is most reactive at 408 nm but remains sensitive to wavelengths between 400 and 430 nm. 19 This spectrum of light is present in *Multipeak* LCUs with wavelength peaks at 405, 440, and 460 nm but not in the *Monowave* LCUs, which commonly present a wavelength peak ∼460 nm. 20 For the other bulk-fill RBCs in which the manufacturer does not mention the initiator used or only CQ is present, the light spectrum emitted from the *Monowave* LCU was sufficient to achieve similar DC to that obtained with the *Multipeak* LCU. The manufacturers of the bulk-fill RBCs used in this study do not completely indicate the specific initiators and the number of initiators used in these materials. The limitation of this test was that the size of the FTIR reading platform only allowed readings to be performed in the center of the specimens, and it was not possible to analyze the DC in peripheral areas.

The hardness of dental materials is an important aspect for the selection of different restorative approaches on posterior teeth. 5 In the present study, no bulk-fill RBCs presented lower KHN values than the convectional composite tested. Only *Aura Bulk Fill* 7-mm specimens light-cured by *Valo* exhibited similar KHN results to the control group, and the other experimental groups exhibited superior KHN in all conditions evaluated. *Filtek One* exhibited higher KHN results compared with the other RBCs, and a possible explanation may be the different monomers and filler composition present in this material ( Table 1 ). Regarding the DC, LCU was the only relevant factor for the *Tetric N-Ceram Bulk Fill* groups.

There is a high demand for Class II restorations, which have an annual failure rate of 1.68% over 12 years. 21 Conventional and bulk-fill RBCs are suitable materials for these restorations. 14 15 22 Clinically, several LCUs present smaller tips compared with the restorative area that needs to be reached by light (10). Mesioocclusodistal (MOD) cavities, such as those noted in first maxillary molars with a 10.31-mm mean MD-D, second maxillary molars (9.79 mm MD-D), and first (6.98 mm MD-D) or second maxillary premolars (6.74 mm MD-D) may present superior dimensions compared with the LCU tip. 11 Thus, the specimens in this study exhibited two different diameters: 7 mm (equivalent to maxillary premolars MD-D) and 10 mm (equivalent to maxillary molars MD-D).

The conventional composite *Amaris* and the *Filtek One* bulk-fill exhibited variations in KHN, which were verified at the central and peripheric regions of the specimens. KHN measurements were performed at the top of the specimens given that the main objective was to verify the influence of the LCU tip diameter and not the polymerization depth. The central region of the *Amaris* and *Filtek One* specimens exhibited increased KHN values compared with the periphery. These results are consistent with previous studies that reported similar findings. 7 23 The *Tetric N-Ceram* and *Aura* bulk-fill RBCs presented similar hardness values at the center and periphery. This fact can be justified by the composition of the organic matrix in these composites that allows greater dispersion of light or the presence of additional initiators that may consequently lead to favorable physical properties in the periphery. 24 25

The *Valo* LCU has four LEDs positioned in the different quarters of the tip diameter, which results in a nonuniform wavelength light beam emission because three LEDs emit blue light (two with peak emission at 460 nm and one with at 440 nm) and one LED emits violet light (peak emission at 405 nm). 20 Despite this fact, no differences in KHN were assessed in the center or periphery of the specimens for the bulk-fill resin composite with the additional initiator (Ivocerin). This finding indicates that the rotation angle of the light tip from multipeak LCUs may not affect the properties of RBCs with different photoinitiators from CQ. The KHN test was performed at the top of the specimens to analyze the possibilities of using bulk-fill composited in wide cavities, allowing a single increment to be used in such situations. This is important, since in the incremental technique, it should be avoided joining antagonistic walls in one increment, such as buccal with lingual and mesial with distal walls. 26

LCUs with small-diameter tips should not be an issue if an incremental filling technique is used. 7 However, reduced light tips may become a problem when a bulk technique is used for extensive MOD restorations. Additional light exposure in the peripheric regions of MOD and larger cavities in posterior teeth is subsequently recommended. 23 Thus, clinicians can assure that all bulk-fill resins receive proper light irradiance, even when using LCUs with small tips. To minimize this problem, additional light exposure in the mesial and distal regions is suggested. LCUs with wide tips and longer exposure times are preferred when light-curing MOD or other large restorations. 23

Despite the limitations of mechanical laboratory tests, they can provide better understanding of fragile materials that are more likely to fail early as RBCs. 27 28 The light beam profile provides information on the irradiance distribution from LCUs, 8 and the light emitted from LCUs influences the polymerization of light-cured RBCs and consequently its properties. 6 Several LCUs present very irregular beam profiles with very high irradiance values at the center of the tip and low values or even no irradiance at the periphery. Thus, the effective light-curing area can be even smaller than the tip of the device. 8 23 Despite this, the mold and the diameter used for preparing the specimens can influence the DC of the composites. As one of the factors analyzed in this study was the restoration dimension (specimen diameter), it was not possible to standardize the diameter between specimens. 29

The distance from the tip of the LCU to the restoration can also influence the irradiance reaching the material and consequently its physical properties. 30 In this study, tests were performed with the LCU in close contact to the RBCs. This condition represent the ideal condition, but there are clinical situations in which it is not possible to place the LCU tip in close contact to the restoration, such as in deep cavities larger than 5 mm and proximal regions with adjacent teeth. 31 In addition, LCUs are generally poorly maintained in dental offices and can deliver inadequate light output. 6 This is a limitation of the present study, as light was always delivered from a favorable position and the LCUs were maintained in ideal conditions.

Therefore, clinicians should be aware that the properties of the restoration are material dependent, and bulk-fill RBCs available on the market may present very distinct physical properties. In addition, it is also important to distinguish the initiators present in the resin composites that are used in routine practice and the emission spectrum of the LCU given that these aspects are important to achieve adequate mechanical properties for RBCs. Unfortunately, some manufacturers do not provide this information. Studies are necessary to further investigate the relationship between the tip diameter of LCUs and the properties of RBCs.

## Conclusion

Within the limitations of the present study, it was possible to observe that the light spectrum of the multipeak LCU significantly increased the DC and KHN of a bulk-fill resin composite with additional initiator to CQ, compared with the monowave LCU. LCU tip size influenced the performance of some RBCs tested. The influence of LCU on the properties of RBCs is material dependent.
